# Supplementary material for: Organic Photodetectors with Extended Spectral Response Range Assisted by Plasmonic Hot-Electron Injection
Source: Nanomaterials (Basel). 2022 Sep 5;12(17):3084. doi: 10.3390/nano12173084 (PMC9458234; doi:10.3390/nano12173084)
Supplement: Supplementary file 1 [file nanomaterials-12-03084-s001.zip › nanomaterials-1877226-supplementary.pdf]

nanomaterials

Supporting information

Organic Photodetectors with Extended Spectral Response Range Assisted by Plasmonic Hot Electron Injection

S1. Information of the material

ITO-coated glass with surface resistance smaller than 10 Ω/sq. was purchased from South China Science & Technology Co. Ltd. Acetone (99.8%) and isopropyl alcohol (99.8%) were purchased from Sinopharm Chemical Reagent Co., Ltd. and Tianjin Zhiyuan Chemical Reagent Co., Ltd respectively. 8-hydroxyquinoline aluminum (Alq_3) with the purity of 99.995% was purchased from Shanghai Aladdin Bio-Chem Technology Co. Ltd. Silver particles (99.99%, Φ~2*5 mm) and tungsten filament were purchased from Zhongnuo Advanced Material Technology Co. Ltd. Aluminum particles (99.999%, Φ ~ 3*3 mm) and tantalum boat were purchased from Fuzhou innovation optoelectronic Technology Co., Ltd. All chemicals were of analytical grade and used without further purification.

S2. Simulation Methods

The simulated configuration is glass/ITO (150 nm)/Alq_3(50 nm)/AgNP/Al(10 nm), and the electric field distributions at 420 nm, 540 nm, 660 nm and 850 nm are investigated theoretically by the three-dimensional (3D) Finite Element Method (FEM). Period (denoted by P) of the simulated configuration was set to 100 nm, and three Ag NPs appeared as semi-ellipsoid were placed in each period. The central localtion of Ag NPs were set to P/6, P/2, 5P/6 respectively, and considering the irregularity of the Ag NPs, minor axis of the middle Ag NP was set to 1.2 times the neighbors, whose major axis and (normal to the film plane) and minor axis (along the film plane) were set to 60 nm and 12 nm, respectively. The schematic for plasmonic resonance oscillations of Ag NP with the shape of semi-ellipsoid is shown in Figure S1. The simulation was carried out assuming periodic boundaries along the x-axis and y-axis, which are normal to the film plane. Perfectly matched layer (PML) boundaries are applied at two planes parallel to the x-y plane, one lies in the glass and the other is in the air region next to the Al layer. Light is illuminated from the glass/ITO side. The wavelength dependent refractive indices and extinction coefficients of the Alq_3 are measured in experiment. The refractive indices and extinction coefficients of other materials used in this work are extracted from Refs. [48, 49].

Simulated results of the electric field |E| for the plasmonic OPD are shown in Figure S2(a), from which, we can see the resonance intensity between the two neighbor Ag NPs changes with the different incident wavelengths. It should be mentioned that the Ag NPs are irregular and vary from each other in size, shape and distance. The simulated results in this work can only approximately give the distribution of the electric field. In order to show the local surface plasmonic resonance of Ag NP more clearly, the single Ag NP with the shape of semi-ellipsoid in a period was also simulated as shown in Figure S2(b). We can see the field exhibits the dipole mode when the Ag NP was illuminated, but light incident with different wavelengths will cause different E field intensity. Compared with Figure S2(a), we can conclude that not only the dipole mode was stimulated but also the hybrid resonances were motivated between the two adjacent Ag NPs. By contrast, in Figure S2(b), there is not any electric field enhancement in the control OPD.

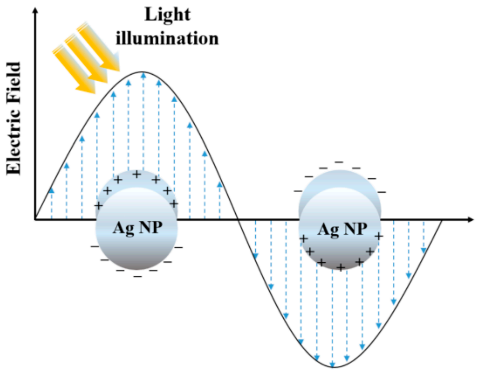

Figure S1 Schematic for plasmonic resonance oscillations of Ag NP with the shape of semi-ellipsoid.

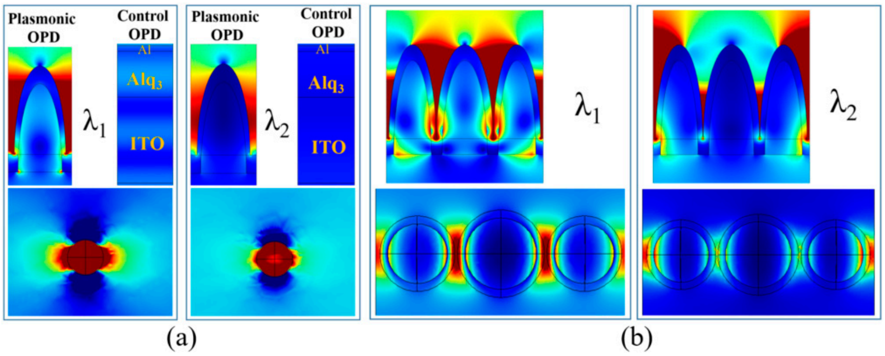

Figure S2 Simulated results of electric field |E| for the control OPDs and plasmonic OPDs with different incident wavelengths. (a) Single Ag NP with the shape of semi-ellipsoid was set in a period. (b) Three Ag NPs with the shape of semi-ellipsoid were set in a period.

S3. Other experimental results

Figure S3 shows the linear I-V characteristics of the control OPD and the plasmonic OPD in the dark, respectively. The control OPD exhibits the unipolar conductivity property in the dark while the plasmonic OPD exhibits a suppressed dark current under forward bias above 2 V.

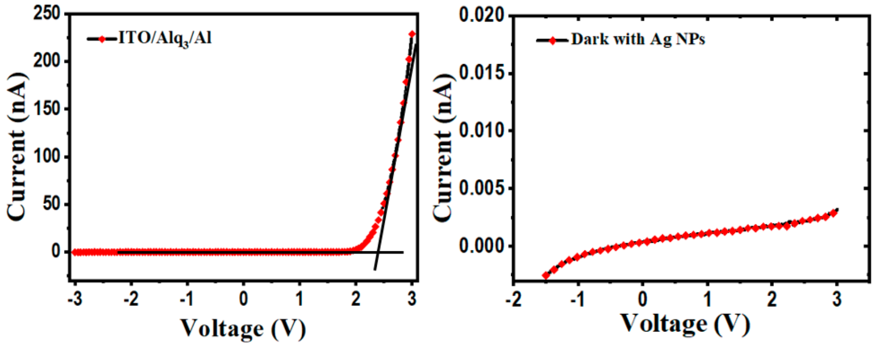

Figure S3. Linear I-V characteristics of the control OPD (a) and the plasmonic OPD (b) in the dark.

The schematic energy level diagrams of the plasmonic OPD at 0 V in the dark is presented in Figure S4. The built-in electric fields that established by the Schottky contacts of ITO/Alq_3 and Alq_3/Ag NPs-Al distributed at limited ranges.

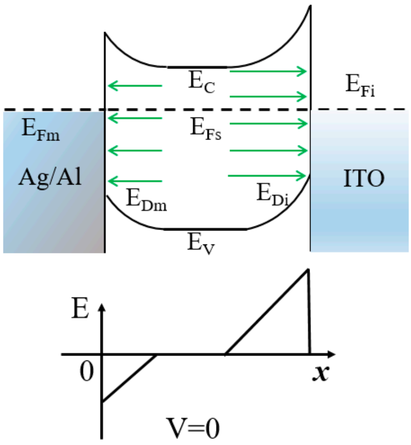

Figure S4. Schematic energy level diagrams of the plasmonic OPD under zero bias in the dark.

Linear I-V characteristics of the plasmonic OPD at 660 nm and 850 nm are displayed in Figure S5. The plasmonic OPD exhibits the unipolar conductivity property under illumination of 660 nm or 850 nm light. When the forward bias is sufficiently high, being above ~1.8 V, the Schottky barrier on the ITO anode side can be fully counterbalanced by the applied voltage, resulting in the smooth flow of carriers from Alq_3 into the ITO anode. The hot carriers excited by the SPPs at long wavelengths can eventually produce electric signals in the external circuit.

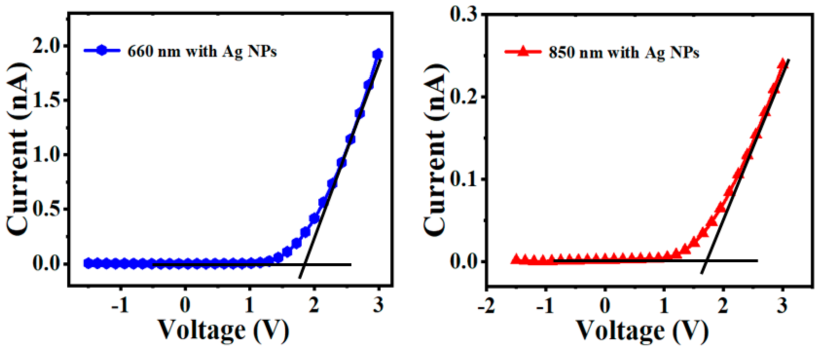

Figure S5. Linear I-V characteristics of the plasmonic OPD at (a) 660 nm and (b) 850 nm.

Negligible differences are observed between the responses of the two OPDs at the wavelength of 375 nm, i.e., the Alq_3 intrinsic absorption band. It is observed that the control OPD can also sense light at 565 nm due to sub-bandgap absorption.

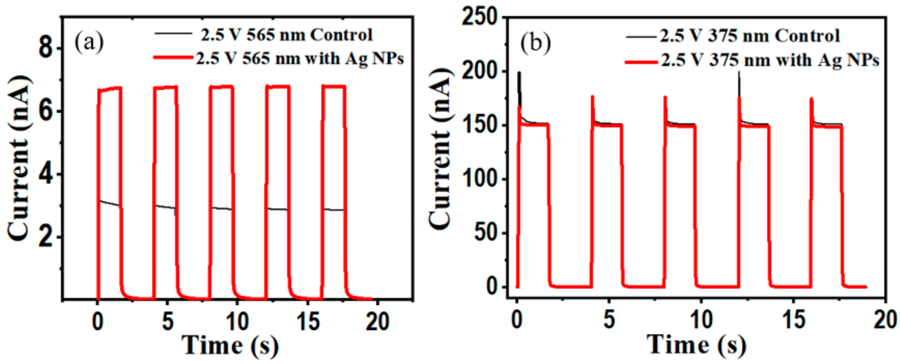

Figure S6. Transient current responses of different OPDs using (a) 565 nm and (b) 375 nm pulse LEDs as illumination sources.

The elemental EDS map of ITO/Alq_3/Ag NPs is presented in Figure S7, from which we can obtain the distribution of Ag NPs. The cyan regions represent Ag element, the brighter the color, the higher content. The black regions represent there is no Ag NPs.

Therefore, we can see, between the island-like Ag clusters, there is a small amount of Ag NPs deposited on the Alq_3 layer in the process of thermal evaporation.

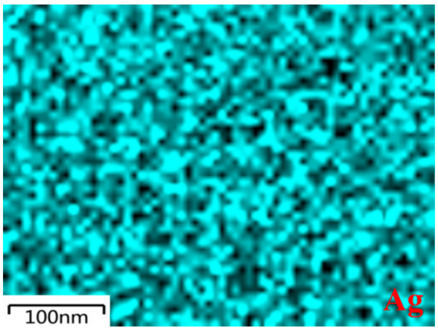

Figure S7 Elemental EDS map of ITO/Alq_3/Ag NPs

The influence of silver thickness on the OPD performance was studied systematically. Figure S8 presents the I-V curves in dark, I-V curves with LED illumination at 850 nm, photo-to-dark ratio, and absorption of the plasmonic OPDs when different thick Ag layers were deposited on the 50 nm Alq_3 layers. From Figure S8(a), the dark currents decreased apparently when Ag NPs were introduced, but the dark currents increased again when the thickness of Ag layers is more than 10 nm. It may be because instead of the Schottky barrier that formed between the Alq_3 and the composite electrode of Ag NP/Al, the organic semiconductor of Alq_3 connected with Ag film directly, leading to a lower barrier and a higher leak current. Figure S8(b) displays the I-V curves when the plasmonic OPDs were illuminated at 850 nm with the incident power density of 45 mW/cm^2. Together with the optimal performance of P/D ration and absorption, from Figure S8(c-d), the most optimized thickness of Ag film is 7.5 nm.

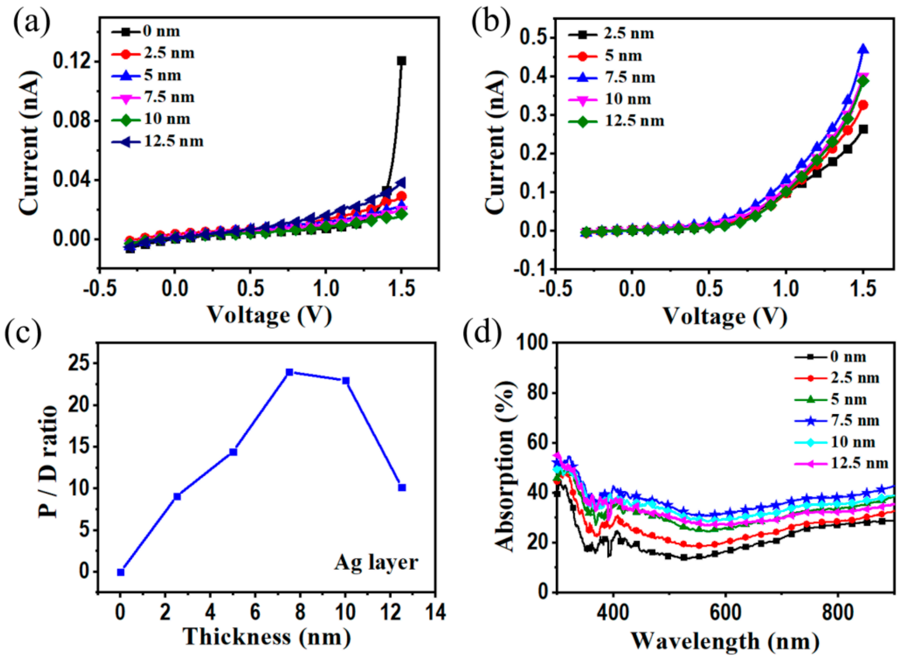

Figure S8 Different thickness of Ag layers performed on the plasmonic OPD: (a) I-V characteristic in dark, (b) I-V characteristic at 850 nm with the incident power density of 45 mW/cm^2, (c) photo-to-dark ratio, and (d) absorptions.

References

48. W. Wang, Y. Cui, K. H. Fung, Y. Zhang, T. Ji, and Y. Hao, Comparison of Nanohole-Type and Nanopillar-Type Patterned Metallic Electrodes Incorporated in Organic Solar Cells, *Nanoscale Res. Lett.* **2017** *12* (1) 538.
49. Z. Wang, Y. Hao, W. Wang, Y. Cui, Q. Sun, T. Ji, Z. Li, H. Wang, and F. R. Zhu, Incorporating silver-SiO_2 core-shell nanocubes for simultaneous broadband absorption and charge collection enhancements in organic solar cells, *Synthetic Met.* **2016** *220* 612-620.
